# Mosquito (Diptera: Culicidae) Fauna of a Zoological Park in an Urban Setting: Analysis of *Culex pipiens s.l.* and Their Biotypes

**DOI:** 10.3390/insects15010045

**Published:** 2024-01-09

**Authors:** Sara Madeira, Rui Bernardino, Hugo Costa Osório, Fernando Boinas

**Affiliations:** 1 CIISA—Centre for Interdisciplinary Research in Animal Health, Faculty of Veterinary Medicine, University of Lisbon, 1300-477 Lisboa, Portugal; fboinas@fmv.ulisboa.pt; 2 Associate Laboratory for Animal and Veterinary Sciences (AL4AnimalS), 1300-477 Lisboa, Portugal; 3 Lisbon Zoo, 1549-004 Lisboa, Portugal; ruibernardino@zoo.pt; 4 CEVDI—INSA—Centre for Vectors and Infectious Diseases Research, National Institute of Health Doutor Ricardo Jorge, 2965-575 Águas de Moura, Portugal; hugo.osorio@insa.min-saude.pt; 5 ISAMB—Instituto de Saúde Ambiental, Faculdade de Medicina, Universidade de Lisboa, 1649-028 Lisboa, Portugal

**Keywords:** *Culex pipiens* complex, *Culex pipiens* biotype, flavivirus, mosquito survey, mosquito-borne diseases, mosquito density, mosquito diversity, urban area, zoological parks

## Abstract

**Simple Summary:**

Mosquitoes are known for being a nuisance but also as important vectors of disease agents that affect not only humans but also animals. Zoological gardens are special places where humans and animals are found in close proximity, and where mosquitoes can also find the conditions required for their life cycle. This can be especially true for zoos located in urban areas. In this study, we characterized, for the first time, the mosquito fauna of Lisbon Zoo, and we found a low mosquito density and diversity. We found an average of 2.4 mosquitos per trap/night, and five different species were identified. The most common species was the northern house mosquito, *Culex pipiens*, with sympatric occurrence of the two biotypes and their hybrids in most collections. Mosquitoes were present year-round, with activity detected in winter months, in which mosquitoes usually diapause. This co-occurrence and activity during winter can have implications in terms of disease transmission, namely, flavivirus, which can affect both animals and humans.

**Abstract:**

Mosquito-borne diseases (MBDs) are important emerging diseases that affect humans and animals. Zoological parks can work as early warning systems for the occurrence of MBDs. In this study, we characterized the mosquito fauna captured inside Lisbon Zoo from May 2018 to November 2019. An average of 2.4 mosquitos per trap/night were captured. Five mosquito species potentially causing MBDs, including *Culex pipiens* biotypes, were found in the zoo. The sympatric occurrence of *Culex pipiens* biotypes represents a risk factor for the epizootic transmission of West Nile virus and Usutu virus. The mosquito occurrence followed the expected seasonality, with the maximum densities during summer months. However, mosquito activity was detected in winter months in low numbers. The minimum temperature and the relative humidity (RH) on the day of capture showed a positive effect on *Culex pipiens* abundance. Contrary, the RH the week before capture and the average precipitation the week of capture had a negative effect. No invasive species were identified, nor have flaviviruses been detected in the mosquitoes. The implementation of biosecurity measures regarding the hygiene of the premises and the strict control of all the animals entering the zoo can justify the low prevalence of mosquitoes and the absence of flavivirus-infected mosquitoes.

## 1. Introduction

Arboviruses (arthropod-borne viruses), which depend on arthropod vectors for transmission, are emerging pathogens in Europe [1]. Mosquitoes are the most important vectors because they transmit mosquito-borne infectious diseases (MBDs), such as dengue and malaria, that can cause high mortality in humans [2]. They are also responsible for the transmission of viruses affecting both animals and humans, such as West Nile virus (WNV), Rift valley fever virus (RVFV), or Usutu virus (USUV), for example [3]. Entomological surveillance can inform us about the risk of MBD transmission and dissemination and work as an early warning for outbreaks. In urban areas, the monitoring of mosquito occurrence is usually carried out in residential areas, cemeteries, or industrialized areas with man-made water containers that can be used as larval habitats, but also in green areas and near ponds and lakes.

Historically, zoos have acted as reliable early warning locations for the introduction of emergent infectious diseases in new areas [4,5,6]. The most recognized example was the case of the WNV outbreak in New York in the United States in 1999, which represented the first incursion of WNV in the American continent and was preceded by the high mortality of wild crows at the Bronx Zoo [7]. 

Zoological gardens are unique collections of microhabitats in which exotic animal species, native fauna, humans, and arthropods can coexist. In these ecosystems, if not controlled, mosquitoes can find optimal conditions for survival: a large range of vertebrate hosts on which to feed, aquatic breeding habitats, and shelter [8]. Mosquito occurrence in zoos can have an impact on the behavior and welfare of captive animals. Mosquito bites are known for being a nuisance, causing irritation and discomfort, and leading to changes in behavior and a decreased appetite. This can affect the overall health and wellbeing of zoo animals and can also have implications in terms of human health. 

Mosquito-borne diseases that affect zoo animals can be of parasitic or viral etiology. 

Avian malaria is the most common parasitic disease in European zoos and mostly affects penguins and owls. In temperate climates, the primary vector is the common house mosquito, *Culex pipiens* [9]. Cases of avian malaria were previously reported in the penguin population of Lisbon Zoo (Bernardino, personal communication, 2018). West Nile virus and USUV are the most frequent causes of zoonotic disease with viral etiology in zoos [10]. The two flaviviruses share many common features, such as the enzootic cycle involving ornithophilic mosquitoes such as *Culex pipiens* and birds. Wild birds act as amplifying hosts, and humans, horses, and other vertebrates are dead-end hosts but can sometimes develop severe neurological disorders. These diseases can be potentially fatal, particularly in animal species that are naïve to these infectious agents [1]. 

The *Culex pipiens* complex includes two of the most widespread mosquito species, namely, *Culex quinquefasciatus* Say, 1823, in tropical and subtropical regions, and *Culex pipiens* Linnaeus, 1758, in temperate regions [11]. The species *Cx. pipiens* consists of two morphologically identical biotypes, *pipiens* Linnaeus 1758 and *molestus* Forskål 1775, which differ in physiology and behavior [12]. Hybridization events between the two biotypes can occur, and the resulting individuals share the behavioral traits of both biotypes, including an intermediate host preference, biting both mammals and birds. Both biotypes and their hybrids are susceptible to WNV and USUV infection and/or have been proven to be competent vectors [13]. This could have an impact in terms of disease transmission, because hybrids can act as bridge vectors between birds and dead-end hosts, including humans [14,15]

The National Vector Surveillance Programme (Rede de Vigilância de Vetores, REVIVE) has been in place since 2008 and has identified 25 species of mosquitoes [16,17] of the Portuguese mosquito fauna, but the mosquito population at Lisbon Zoo has never been examined.

In this study, we aimed to characterize the mosquito fauna present at Lisbon Zoo, including the identification of *Culex pipiens* biotypes, as well as screening mosquitoes for flaviviruses, in order to characterize the potential epidemiological role of the zoo. 

## 2. Materials and Methods

### 2.1. The Study Area and Mosquito Sampling

Mosquito collections were performed at Lisbon Zoological Garden (centroid: lat 38.74452, long −9.17072), which is located in Lisbon City Center, within an urban area, surrounded by residential buildings, and with a large urban park (Monsanto) located at a linear distance of approximately 700 m and the Tagus River at a linear distance of five kilometers (map available in Supplementary Data, Figure S1). The Lisbon region has a temperate continental dry-summer climate (Csa in the Köppen–Geiger classification) that is mild with moderate seasonality (https://www.lisbon.climatemps.com/, (accessed on 30 October 2023)).

Lisbon Zoo hosts approximately 2000 animals of more than 300 species, in an area of 22 ha. The landscape is characterized by deciduous and coniferous trees, with artificial ponds throughout the park.

Adult mosquitoes were collected from May 2018 to November 2019, every two weeks, using three Center for Disease Control (CDC) miniature light traps baited with dry ice as a source of carbon dioxide (CO_2_), which were operated from dusk to dawn. The trapping frequency was selected taking into account the European Center for Disease Control (ECDC) guidelines for the surveillance of native mosquito species [18], as well as previous works in zoos [19,20,21]. The traps were placed from 1.7 to 2 m high. The traps were installed in three permanent locations inside the zoo, at least 100 m apart: outside the premises of avian species (birds and penguins), zebras (equids), and giraffes (ruminants) (Supplementary Data, Figure S2). The criteria for site selection were related to host proximity, cover, and protection of the traps from the sun and rain, the safety of the animals and people, and logistics. After collection, mosquitoes were transported to the laboratory and immediately stored at −20 °C and, within a week, species identification was performed.

### 2.2. Mosquito Identification

Mosquitoes were killed via cold in a −20 °C freezer and then kept dry on a chill table during morphological identification. Identification was conducted via individual observation under a stereoscope, using the identification keys of Ribeiro and Ramos [22] and Schaffner et al. [23] and mosquitoes were subsequently stored at −80 °C. 

A sub-sample of *Culex pipiens* was used for molecular identification of their biotypes. Up to five *Cx. pipiens* were selected from each capture (trap/night) and individually subjected to DNA extraction (see Section 2.3) and to a multiplex PCR to detect a polymorphism in the flanking region of the CQ11 microsatellite [24]. 

### 2.3. Nucleic Acid Extraction

For DNA extraction, *Culex pipiens* specimens were individually ground in 1 mL of cell culture medium (Minimum Essential Medium (MEM), GIBCO™, Thermo Fisher Scientific^®,^ Waltham, MA, USA) using glass beads and a homogenizer (Disruptor Genie^®^, Scientific Industries, Inc., New York, NY, USA) for 10 min. Half the volume of the resulting homogenate was then used for DNA extraction using a column-based extraction kit (DNeasy^®^ Blood and Tissue Extraction Kit, Qiagen^®^, Hilden, Germany) or an automatic extractor (Nuclisens^®^ easyMag^®^, BioMérieux, Boxtel, The Netherlands), which extracted DNA and RNA in one single run. The extracted DNA and RNA were stored at −20 °C and −80 °C, respectively, until further processing. 

For viral detection, female specimens of each species were subject to RNA extraction. Samples were processed in pools of a maximum of 50 individuals, consisting of females belonging to the same species. This resulted in eight pools, each containing between three and forty-seven female mosquitoes, as described in Table 1.

Mosquito pools were ground with liquid nitrogen in a cold mortar using a pestle and homogenized in 1 mL of MEM. Half the volume of the suspension was then centrifuged in a homogenizer cartridge (Invitrogen™, Thermo Fisher Scientific^®^, Waltham, MA, USA) and subjected to RNA extraction using either an automated protocol in EasyMag or a column-based extraction kit (Ambion™ PureLink™ RNA Mini Kit, Invitrogen™, Thermo Fisher Scientific^®^, Waltham, MA, USA), according to the manufacturer’s instructions. All steps were performed in a laminar flow cabinet, and all reagents were kept on ice during the procedure.

### 2.4. Viral Detection

RNA pools were subjected to reverse transcription polymerase chain reaction (RT-PCR) for pan-flaviviruses, targeting a conserved region of approximately 200 bp of the NS5 gene. The primers used were EDL/Fla-U9093 (forward), 5′- AGY MGR GCH ATH TGG TWY ATG TGG -3′ and EDL/Fla-L9279 (reverse), and 5′- TCC CAV CCD GCK GTR TCA TC -3′ at 10 µM [25,26]. For the reverse transcription (RT) reaction, a one-step commercial kit was used (SuperScript™ One-Step RT-PCR with Platinum™ Taq, Invitrogen™, Thermo Fisher Scientific^®^, Waltham, MA, USA), for a final reaction volume of 20 µL, with the following PCR protocol: a first step of cDNA synthesis at 50 °C for 30 min, followed by initial denaturation at 95 °C for two minutes. Next, 40 cycles of denaturation at 94 °C for 15 s, annealing at 60 °C for 15 s, and extension at 72 °C for 15 s were performed, with a final extension performed at 72 °C for 5 min. For the “nested PCR” reaction, a high-fidelity PCR master (FastStart^®^, Roche, Mannheim, Germany) was used for a final volume of 25 µL, under the same conditions as previously described. Positive and negative controls were used for each reaction. Positive controls consisted of previously isolated flaviviruses, kindly supplied by INSA/CEVDI: either tick-borne encephalitis virus, dengue virus, or WNV. The amplification products were observed on a 2% agarose gel stained with GelRed^®^ (Biotium, Fremont, CA, USA).

### 2.5. Mosquito Diversity and Abundance Modelling

Statistical analyses were performed using R software (version 4.1.0, R core team, Vienna, Austria) [27] and the R Studio interface (version 1.3, Boston, MA, USA) [28].

Species diversity indices were calculated. Simpson’s diversity index reflects the probability that two individuals taken at random from the dataset are not the same species, and its values range between 0 and 1, with larger values representing greater diversity. The Shannon–Wiener diversity index, for which larger values represent greater diversity, was also calculated.

To investigate whether a sufficient sampling effort was made for a correct estimation of species diversity, a species accumulation curve of the number of collected mosquitoes was designed using the VEGAN (version 2.5-7) and iNEXT (version 2.0.20) [29] packages.

Mosquito abundance was calculated as the number of mosquitoes captured each week (sum of the captures from the three habitats). Differences between the total number of mosquitoes captured each year and month were analyzed using the chi-squared test of homogeneity.

To evaluate the factors that affect the abundance of *Culex pipiens*, a generalized linear model (GLM) was fitted. We used the number of mosquitoes of this species (count data) as the response variable, and weather variables were used as independent variables. Data from the closest weather station, located at approximately 4 km (also located in an urban area), were used (Supplementary Data—Figure S1). Daily weather variables such as temperature (minimum, average, and maximum), precipitation, relative humidity (RH), or wind speed were downloaded from OGIMET Weather Information Service [30], using the ‘Climate’ package (version 1.0.5) [31]. Weekly averages of the different variables were used, except for precipitation, for which the sum of the total precipitation for each week was calculated.

Weather variables can affect mosquito abundance not only as daily variables, but also as variables with a time lag in relation to the date of capture. This is mainly due to the influence of environmental and climatic variables on the availability of aquatic habitats for the immature stage, as well as the rate of development of immatures and activity of adults, which, as a consequence, affect adult mosquito abundance [32,33]. As mosquito development from egg to adult takes approximately 7–10 days, we considered that a time lag of one week would better describe the weather variables that could have a biological effect on mosquito development and adult mosquito activity. The computed different time-aggregated variables that were included in the GLM are described in Supplementary Data, Table S1.

As these variables can be strongly correlated, in order to account for multicollinearity, we used variance inflation factor (VIF) to select the variables to use on the GLM, eliminating sequentially the variables with higher VIFs, until all variables had VIFs lower than four [34]. A Poisson distribution with log link was first tested, but as the model was overdispersed, a negative binomial distribution was used instead, using the package MASS [35]. A stepwise backward elimination procedure based on *p*-value (significance level *p* = 0.05) was used for variable selection, and AIC ‘evaluation’ was used for model comparison.

## 3. Results

### 3.1. Number of Collections/Captures

During this study, mosquito collections were performed twice a month at the three permanent sampling locations in the zoo. This means that in each month, six captures were performed, except for October 2019, in which the three traps were operated only once a month. This corresponds to a capture effort of 37 trap nights and 111 mosquito collections performed from May 2018 to November 2019, a 19-month period. However, 6 out of the 111 collections were not valid, due to malfunctions of the traps, resulting in a total of 105 valid mosquito collections. In 2018, a total of 44 valid captures were performed during the eight-month sampling period, while in 2019, during the 11-month sampling period, 61 valid captures were performed, with an average of 5.5 valid captures per month in each year.

Fifty-four percent (N = 57) of valid collections captured mosquitoes, while null captures (captures with zero mosquitoes, but where other *Diptera* were trapped, indicating that the traps were operating properly) accounted for 46% (N = 48).

### 3.2. Mosquito Abundance

The valid collections allowed the capture of 251 mosquitoes, representing an average of 2.4 mosquitos per trap per night. Of the total mosquitoes sampled, 66.5% (N = 167) were females, and 33.1% (N = 83) were males (χ^2^ = 24.934, d.f. = 1, *p*-value < 0.0001). One gynandromorphic specimen was also collected. No visibly blood-fed females were collected in this study.

Significant differences were detected between the total number of mosquitoes captured in each year of collection (χ^2^ = 6.3249, d.f. = 1, *p*-value = 0.01191), with 58.2% (N = 146) of the mosquitos being captured in 2018 and 41.8% (N = 105) in 2019. To account for the different number of sampling months in each year, we also compared the average mosquito abundance by number of captures, with 3.3 mosquitoes per capture in 2018 and 1.7 mosquitoes per capture in 2019.

**Figure 1:**
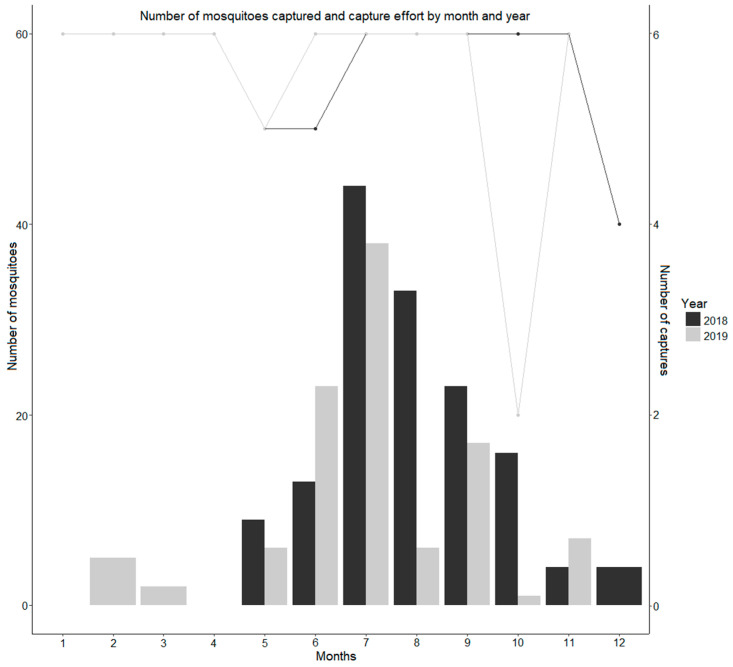
Seasonality of mosquito occurrence and capture effort by month and year. The number of mosquitoes is represented by the graph bar and can be measured on the y axis. The capture effort is measured by the number of captures, which is represented by the lines in the graph and can be read on the accessory vertical axis. The capture effort was six captures per month, except for October 2019, during which three captures were performed. The number of valid captures was also affected by trap malfunction.

The distribution of mosquitoes among months followed the expected seasonality, with increasing numbers from May to July, with a monthly maximum of 44 and 38 mosquitoes in 2018 and 2019, respectively, and a decreasing trend until December (Figure 1), although in 2019, we can identify a sudden decrease in the number of mosquitoes captured during August, followed by an increase in September, and then a new drop in mosquito abundance. 

### 3.3. Species Richness

The total species richness for the zoo was five species—*Culex pipiens*, *Culiseta longiareolata*, *Cx. theileri*, *Aedes caspius*, and *Cs. annulata*—with a Shannon index of 0.721 and a Simpson’s index of 0.398. The accumulation curve shows that the sampling effort was adequate for the correct determination of species richness, as the curve is beyond its exponential growth, reaching an asymptote (Supplementary Data—Figure S3). This means that even if the sampling effort had been extended, there should have been no more different species captured.

*Culex pipiens* was the most abundant species, accounting for 73.3% (N = 184) of the mosquitoes captured, followed by *Cs. longiareolata*, at 21.2% (N = 53). Other species had only sporadic occurrences, such as *Cx. theileri* (N = 5; 2%), *Ae. caspius* (N = 3; 1.3%), or *Cs. annulata* (N = 2; 0.8%), with only female specimens captured. No exotic species were detected in the samples.

### 3.4. Culex pipiens Abundance and Seasonality

Although it was always the most common species reported, the proportion of *Cx. pipiens* among the total mosquitoes captured varied between the two years: in 2018, *Cx. pipiens* corresponded to 81.5% of the total mosquitoes captured, while in 2019, this number decreased to 62%. The opposite occurred with *Cs. longiareolata*, while in 2018, this species corresponded to 11% of the captures; in 2019, it represented 35% of the total of mosquitoes captured.

Overall, the *Cx. pipiens* number increased from May to July, when the prevalence reached its maximum, both in 2018 and 2019 (N = 41 and N = 23, respectively) and maintained high densities (>30 mosq/month) in August and September. From then on, the density dropped to almost half, but in 2019, this drop was more dramatic than in 2018. In other months, densities were below 20 mosq/month, with zero specimens captured in January and April, but in February and March, some activity was detected. 

### 3.5. Factors Affecting Number of Culex pipiens—Weather Variable Modeling

The differences found between years in terms of species occurrence, density, and pattern of seasonality led us to investigate the factors that could explain this distribution. A GLM using weekly averages of weather variables as covariates and the number of *Cx. pipiens* was used.

Analysis of VIFs for the detection of multicollinearity between weather variables led to the elimination of correlated variables, and the GLM model included the variables: ‘average precipitation on the week before capture’, ‘average RH on the week before capture’, ‘average precipitation on the week of capture’, ‘average RH on the week of capture’, ‘average wind intensity on the week of capture’, ‘precipitation on the day of capture’ (yes or no), ‘wind intensity on the day of capture’, ‘RH on the day of capture’, and ‘minimum temperature on the day of capture’. 

The *Culex pipiens* abundance was positively related to the minimum temperature and the relative humidity on the day of capture, and negatively influenced by the relative humidity on the week before capture and the average precipitation the week of capture (Table 2).

### 3.6. Culex pipiens Biotypes

Of the 184 *Cx. pipiens* identified, 51% (N = 94) were subject to CQ11 amplification for biotype identification.

The results revealed that there was an equivalent proportion, with no significant differences, of the two biotypes and their hybrids: *Cx. p. pipiens* accounted for a slightly higher percentage (34.04%) than *Cx. p. molestus* or the hybrids of these two forms (both 32.98%) (χ^2^ = 0.021, d.f. = 2, *p*-value = 0.989). However, the proportion of biotypes and their hybrids was different in each year. While the relative proportion of biotypes *pipiens*/*molestus*/hybrids was 37/24.1/38.9% in 2018, in 2019, these values were 30/45/25%, respectively.

**Figure 2:**
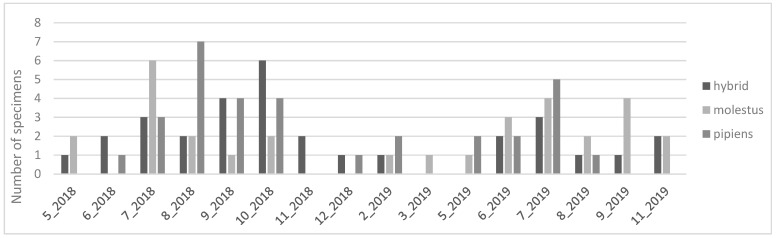
Number of *Culex pipiens* biotypes per month.

The biotype seasonality also demonstrated differences, as shown in Figure 2. While biotype *molestus* had its maximum abundance in July, and a decreasing trend from thereon until November, biotype *pipiens* had its maximum abundance in July and August and was only detected until October. Hybrids of the two forms had a less expressive seasonality, with abundance being constant throughout the year.

When analyzing the *Cx. pipiens* biotypes captured during winter months, all biotypes were detected between December 2018 and March 2019. Although in very low numbers, three specimens of biotype *pipiens* were captured in December and February, and two specimens of *pipiens* x *molestus* hybrids were also present in the same months. Two specimens of the biotype *molestus* were captured in February and March 2019.

### 3.7. Flavivirus Screening

Eight pools of mosquitoes were tested for flavivirus RNA, all with negative results.

## 4. Discussion

Although zoos have been recognized as unique areas for the study of mosquito ecology and as important areas for monitoring MBDs, previous studies in zoos in Portugal have focused mainly on screening for parasitic diseases in animals [36,37], and their mosquito fauna has never been characterized or screened for viral diseases. 

This work is the first characterization of the mosquito fauna associated with a zoological garden in an urban setting in Portugal. We found that native species occur in the zoo, namely, the two biotypes of the widespread species the northern house mosquito, *Culex pipiens*, as well as hybrids of the two forms. The high rate of hybridization reported can have an impact on the risk of flavivirus transmission if other factors are also present.

Entomological surveillance is the primary tool for the risk assessment and management of vector-borne diseases. The main purpose of entomological surveillance programs is to collect data on vector population composition, distribution, and abundance, as well as the quantification of virus infection rates in those populations. This provides indicators of the threat of virus transmission and identifies geographical areas of high risk, supporting decisions around the need for intervention activities [38]. 

In this context, the REVIVE program has been performing mosquito captures, mostly in habitats associated with humans, with country-wide coverage. Adult mosquito sampling is performed using CDC traps or BG-sentinel traps, and mosquito attractants such as CO_2_ (using dry ice or other CO_2_-releasing baits) or octenol, are commonly added. Mosquitoes are subjected to pan-flavivirus screening, but pathogenic flavivirus have never been detected, only insect-specific flaviviruses [39]. The national average of mosquitoes per trap per night reported in 2018 and 2019 was 8.94 and 3.79, respectively. The results for the county of Lisbon are not available, but the regional average for the 28 counties in the NUTS area of Lisbon and Tagus Valley (LVT) was 2.33 and 1.15 mosquitoes per trap per night in 2018 and 2019, respectively. As part of the REVIVE program, mosquitoes were collected at a zoo in a city in northern Portugal, Maia, but the results have only been analyzed at the municipality level, and not specifically for the zoo [39]. The mosquito density for Lisbon Zoo, with an average of 2.4 mosquitoes per trap/night (3.3 and 1.7 mosquitos per trap/night in 2018 and 2019, respectively), is at the same order of magnitude as the average for the LVT area, but is lower than the national average, although with no significant differences. Captures in the LVT area were not as frequent as in the zoo, and in some cases, the trap and bait used can be different from those used in this study, meaning that comparison between the mosquito densities is not straightforward. The same applies when comparing mosquito densities in other zoos. Studies on mosquito fauna composition and ecology in zoos in Europe have used different sampling designs, namely, the types of traps (e.g., BG sentinels, EVS traps, or aspirators) and sampling frequencies, as well as the zoo locations in urban, peri-urban, or rural areas, which makes it hard to make direct comparisons. Overall, the mosquito density reported has been similar [40] or higher than in this study [19,20]. Some authors have emphasized that zoos have attractive characteristics for mosquitoes, such as a high availability of aquatic habitats and feeding opportunities [8,40,41]. Animal drinking troughs, enrichment structures in the animal enclosures, water pits, or decorative water structures around the zoo are commonly described as optimal for mosquito development [8,10]. For these reasons, it might be expected that the mosquito density inside the zoo would be higher. An explanation for this low mosquito density could be due to the biosecurity measures implemented by Lisbon Zoo. Some of the biosecurity measures include daily cleaning of the outside and inside enclosures of all genera of animals (antelopes, primates, felines, etc.), including weekly or twice a week, and the changing and cleaning of pools (rhinos, hippopotamus, elephants) and waters that make up parts of the animal enclosures. Some of the waters are treated with chlorine, and the disinfection of inside enclosures is frequent or daily (for concrete surfaces) using hypochlorite and other products when needed. This makes the zoo a much more controlled environment in comparison to other potential breeding sites in public spaces, such as urban parks and gardens. This study would have benefited from mosquito trapping being performed outside the Zoo, for comparison. This could not be carried out because trap security could not be guaranteed. The REVIVE results show that the mosquito density in the Lisbon area was also considered low, although there is no specific mosquito control program implemented in the Lisbon area (Isabel Marques, Personal Communication). Also, REVIVE found a lower prevalence of mosquitoes in urban areas compared with peri-urban and rural locations [17,39]. 

In terms of species diversity, REVIVE has already reported the occurrence of 25 species in the country, although several of them are rare, having only sporadic and localized occurrences [42]. Comparatively, the species diversity found at the zoo can be considered low, but it is in accordance with the species diversity reported for the Lisbon area. In the LVT region, in 2018 and 2019, the same five species that were detected in the zoo were also reported, as well as an extra species, *Cx. univitattus*, with sporadic occurrence. Also, the low species richness does not seem to be associated with the sampling effort, as demonstrated by the species accumulation curves. Martinez de la Puente et al. (2020) also found limited species diversity at Barcelona Zoo, with only three different species reported [20]. *Culex pipiens* was the most prevalent species (approximately 53%), and *Cs. longiareolata* represented approximately 12% of all adult catches (as the third most prevalent species). On the contrary, Hernadez-Colina et al. (2020) reported a higher species diversity, with 11 species captured [40]. Heym et al. (2018) also found a much higher diversity of species, with as many as 16 different taxa, in the two zoos investigated, as well as higher mosquito densities, but this higher diversity and density were mainly due to the captures in a zoo located in a rural area [19]. Several studies indicate that mosquito diversity (and density) is higher in rural areas than in urban or peri-urban locations [43,44]. Although a low species diversity was found in the zoo, all five species found at Lisbon Zoo are recognized or potential vectors of pathogens of medical and veterinary importance, such as arbovirus, namely WNV, USUV, Japanese Encephalitis virus [45], and parasites, such as *Dirofilaria* or *Plasmodium* spp. This needs to be taken into account, because if the number of mosquitoes increases significantly, and there is the introduction of a pathogen, then the zoo and the Lisbon area could be at risk of disease transmission.

CDC traps baited with CO_2_ tend to attract mostly blood-seeking females, but male mosquitoes were also captured, representing approximately 33% of the total mosquitoes captured. The presence of males can be an indication that the mosquito population has its breeding site inside the zoo, in or close to the sampling site, because the estimated flying distance of adult (irrespective of sex) *Culex* mosquitoes ranges between 0.16 km and 1.98 km [46].

The two-year sampling period enables seasonal comparison between years. Even though the first year (2018) corresponds to eight months of sampling, the total number of mosquitoes captured was significantly higher than the eleven-month sampling period of the second year (2019). Hernandez-Colina and colleagues also found a decrease in the number of females captured by BG traps at Chester Zoo from 2018 to 2019 [40]. 

The abundance of *Culex pipiens* also followed the same trend, with a significant decrease from 2018 to 2019, while *Cs. longiareolata* increased from 2018 to 2019. These two species are commonly found together, as they share the same type of preference regarding aquatic habitats for reproduction [39]. This can lead to competition for the same type of aquatic habitats, so when one is higher, the other is necessarily lower. Another explanation may be related to the distinct climatic variables that affect the two species differently. 

Weather abnormalities could explain the differences in mosquito densities reported here, as mosquitoes are strongly influenced by climatic conditions, mainly temperature, humidity, and precipitation [47]. The year 2018 was an unusual year due to above-average rainfall early in the season and relatively higher spring and summer temperatures [48]. These climatic factors were recognized as important drivers of the extraordinary number of WNV outbreaks in Europe that year [49,50]. On the other hand, the year 2019 was considered as the second hottest year on record globally, and the hottest year recorded for Europe until 2022 [48]. This year was characterized by drier conditions than average, with lower-than-average values for precipitation, surface air relative humidity, and soil moisture [48]. 

In fact, our results show that *Cx. pipiens* were positively influenced by the relative humidity and minimum temperature on the day of capture. It is known that there is a threshold temperature related to mosquito activity, and that small decreases in this minimum temperature can reduce the flight activity of mosquitoes and reduce the emergence of adult mosquitoes from their aquatic breeding habitats [51,52]. 

The mechanism of overwintering or diapause, in which mosquitoes suspend their development, allows them to survive adverse winter climates. For *Culex* species in temperate climates, mosquitoes overwinter as adult females, and can be found in their resting sites inside protected environments or hibernacula, which are commonly anthropogenic [53]. In this study, from December 2018 to May 2019, there were a total of 11 adult females captured in the zoo. As these captures were not performed inside human-constructed shelters, this shows that there is still mosquito flight activity even in winter months, although in a low number. To better understand the winter activity of mosquitoes at the zoo, trapping should be complemented, for example, by the use of gravid traps and a more intense trapping frequency. Also, a higher number of traps, in complementary non-permanent locations at the zoo, during winter months, would have given more information on the overwintering behavior of *Culex pipiens*. 

These significant annual variations and the inexistence of a marked diapause suggest that the mosquito monitoring should continue all year round and for several years, in order for us to better understand patterns and to be able to build reliable predictive models for future occurrence, especially considering climate-change scenarios. Another option is to monitor weather variables and include them in the design of the entomological surveillance program.

In this study, we screened the *Cx. pipiens* population in the zoo for the presence of the *Cx. pipiens* biotypes: biotype *pipiens*, biotype *molestus*, and hybrids between these two forms. 

Historically, the biotype *pipiens* is commonly described as ornithophilic (preference for feeding on birds), exophagic (feeds outdoors), exophilic (rests outdoors), eurygamous (mates outdoor in swarms), anautogenous (needs a bloodmeal for laying eggs), heterodynamic (diapauses during winter), and having a preference for aboveground habitats across urban and rural areas [12,14,54,55]. On the other hand, the biotype *molestus* is described as mammophilic (a preference for feeding on mammals), endophagic (feeds indoors), endophilic (rests indoors), stenogamous (is able to mate in confined spaces), autogenous (does not need a bloodmeal for laying eggs), homodynamic (does not diapause and can be found actively blood feeding during winter), and associated with urban underground breeding habitats [14,56]. Hybrids between the forms tend to show intermediate characteristics, such as an opportunistic feeding behavior, biting both birds and mammals [14]. 

Some of these ecological characteristics have been investigated and are now thought to be more flexible than previously assumed [57]. For example, in cases of host availability, the *molestus* biotype can change its host-feeding preference to birds [58]. Moreover, it was previously thought that the habitat distinction served as a reproductive barrier between the two forms and hybridization was uncommon [59], but there have been several descriptions of sympatric occurrence of both biotypes and their hybrids at different breeding sites in Europe (Portugal [60], Netherlands [61], Italy [62]) and North Africa (Tunisia [57] and Morocco [63]). 

This sympatric occurrence may be related to the milder winters, frequent in Southern Europe, that allow both ecotypes to thrive aboveground, as opposed to higher latitudes, in which there seems to be a separation between biotypes [64]. The natural co-occurrence of both biotypes in open and aboveground habitats favors mating between the forms and the emergence of hybrids [60,61,62,65,66,67,68]. 

It also seems that the preference of biotype *pipiens* for the open, outdoor environments common in farm and natural habitats, as well as the preference of biotype *molestus* for the confined and indoor environments commonly associated with urban habitats, is not so strict. 

Vogels and colleagues found that the proportion of the biotypes *pipiens* and *molestus* were different across three habitats (peri-urban, farms, and wetlands), but these proportions were different in the three countries studied [64]. There are several countries in which *pipiens* and *molestus* biotypes co-occur in urban, peri-urban, and rural habitats [60,62,69]. This can be related to differences in climate, microhabitat, availability of breeding sites, and hosts. 

In Portugal, sympatric occurrence of the different biotypes has been reported, with hybridization rates ranging from 8% to 17.5% depending on the location, with a higher presence of the *molestus* biotypes in urbanized environments, and a negative association between the frequency of *pipiens* and the degree of urbanization [60]. 

Almost all the collections in this study have shown that sympatric occurrence of *pipiens* and *molestus* biotypes is a common occurrence in the zoo. Crossbreeding between the two forms seems to be a very frequent event, as suggested by the high frequency of hybrids (approximately 33%). 

The high hybridization rate found at the zoo can be explained by the fact that in the zoo, mosquitoes can find habitats that promote the occurrence of both biotypes aboveground. The special design of zoos, such as a landscape of non-continuous human buildings, the presence of a higher variability of animal species, and with a high density and diversity of flora, can act as temperature buffers that can mimic the temperatures associated with the mild winters described by Vogels and colleagues [64]. 

The differentiation of biotypes is important because these differences in behavior, physiology, and ecology can have an impact on the vectorial capacity of *Cx. pipiens* populations. *Culex pipiens* populations that are dominated by the biotype *pipiens* play an important role in the natural transmission cycle of these flaviviruses in birds. In contrast, a high prevalence of hybrids contributes to the epizootic cycle, as these individuals can act as bridge vectors, biting both birds and mammals, which can increase the risk of flavivirus outbreaks in humans or other dead-end hosts, such as equids [14,15]. 

The high proportion of the biotype *molestus* and hybrids in the zoo could be of concern because, besides the different host preference, *molestus* and hybrids also have other behavior and physiological traits that can enhance their vector capacity, such as the occurrence of autogeny, the ability to forgo diapause and continue reproduction through the winter months [59].

From December to March, all three biotypes of *Culex pipiens* were captured in the zoo. Although they are described as entering a diapause during winter, we have identified two specimens of the biotype *pipiens* in this season. Even though the number is low, we can speculate that biotype *pipiens* populations can find, in zoos, favorable conditions that enable their activity even during winter, probably related to the availability of microhabitats and breeding sites, and ideal environmental conditions such as temperature. The non-diapausing biotype *molestus* was also found in aboveground habitats during the winter months of February and March 2019. This could be related to the milder winter temperatures that occurred in that atypical year that allowed the survival of biotype *molestus* aboveground in open space, as suggested by Vogels et al., 2016 [64]. This longer period of activity of *Culex pipiens* could be a risk factor for flavivirus transmission. 

There are several factors that influence the risk of flavivirus transmission, and they can be related to the host, the agent, the vector, and the environment. In terms of vectors, the biological transmission mechanism requires a high density of competent vectors, a high vector-survival rate, and frequent contact between the vectors and vertebrate hosts [70]. The definition of a threshold for mosquito abundance that represents a risk for human transmission depends on several factors (such as the mosquito biting rate, mosquito host preference, etc.), but authors agree that arboviral disease outbreaks are associated with high mosquito abundances. Vogels et al. suggest that mosquito densities would have to be higher than 2000 mosquitoes for sufficient WNV mosquito transmission, based on collections performed in Italy. Moreover, Calzolari and colleagues report that the threshold for human cases is a monthly average of 300 *Cx. pipiens* specimens per trap night [71]. The mosquito density found in the zoo is at least 100 times below the suggested thresholds, so the risk of human outbreak, in the case of virus circulation, is negligible. There are no complaints from the zoo workers or the public regarding the nuisance of mosquitos (Bernardino, personal communication). Moreover, the risk of introduction of pathogens through the introduction of infected animals is thought to be low because the origin of the animals is strictly controlled and the animals that are introduced into the zoo are subjected to quarantines and health checks, similar to what happens to livestock under the European Health directives.

Furthermore, no flaviviruses were detected in the mosquitoes captured in the zoo. Although all captured mosquito females were subject to flavivirus screening in pools, if we had a greater number of mosquitoes captured and screened for flavivirus, this would have been valuable. When the disease prevalence is low, as is the case for WNV in Portugal, the sample size needs to be larger in order to detect infection. Despite this, the non-detection of viral circulation in the mosquitoes captured at the zoo was expected. Although, since 2004, four human WNV infections have been reported for Portugal, and serological and molecular evidence of WNV circulation from animals has been proven, the surveillance of flavivirus in mosquito vectors performed by REVIVE since 2008, with an average of 237 pools tested every year, has not detected medically important flaviviruses in mosquitoes [17,72]. Nevertheless, WNV had been previously detected in mosquitoes in Portugal, in 1972 [73] and 2004 [74]. However, in other countries, arbovirus circulation has been detected in mosquitoes, including those captured in zoos [75]. 

There have been no reports of WNV infection in either human or equids in the Lisbon area in recent years, although there was evidence of the circulation of WNV in two birds in a wild bird recuperation center in Lisbon in 2021 [76], so the possibility of circulation in mosquitoes and possible transmission to other hosts should not be neglected.

Contrary to other studies in zoos, in this study, invasive species were not detected at Lisbon Zoo. These species are known for their preference for urban settings, so zoos in urban locations should be prepared. The invasive species *Aedes aegypti* was detected on the Madeira Islands in 2005, where it was responsible for an important outbreak of dengue in 2012, and where it is still present, although in low numbers [77]. *Aedes albopictus* was first introduced to Portugal in 2017 in the north region, and three other introductions were detected: in 2018 in the Algarve region, in 2022 in the Alentejo region [78], and in September 2023 in Lisbon [79]. The targeted monitoring of *Aedes* species is crucial for early detection and strategic control interventions to avoid species establishment in the territories, particularly under the climate-change scenario.

To better understand the risk of mosquito population establishment inside the zoo, future works should include the collection of immature stages. Sampling strategies should also be adapted to increase the collection of blood-fed females, using, for example, gravid traps, resting boxes, or aspirators. The identification of mosquito host preferences would be valuable in understanding the transmission risk of mosquito-borne diseases.

## 5. Conclusions

This study has characterized the mosquito population in a zoo located in an urban area. The mosquito density and diversity were low, and flavivirus infection in the mosquitoes was not detected, probably due to the biosecurity measures implemented in the zoo. However, the native species *Culex pipiens* biotypes and their hybrids were detected all-year round, including during winter months. This can be related to local environmental and ecological characteristics and should be taken in consideration when planning the surveillance of mosquito-borne diseases, namely, flavivirus.

## Figures and Tables

**Table 1:** Number of female mosquito specimens per pool for flavivirus screening.

Pool Number	Number of Specimens	Species
1	3	*Aedes caspius*
2	3	*Culiseta annulata*
3	3	*Culex* spp.
4	5	*Culex theileri*
5	14	*Culiseta longiareolata*
6	46	*Culex pipiens s.l.*
7	47	*Culex pipiens s.l.*
8	47	*Culex pipiens s.l.*

**Table 2:** Weather variables that affect *Culex pipiens* abundance in the zoo.

Response Variable	Explanatory Variable	Coefficient (±S.E.)	*p*-Value
Number of *Culex pipiens*	Intercept	−2.90 ± 1.4	0.04
	Average relative humidity the week before capture	−0.03 ± 0.01	0.04
	Average precipitation the week of capture	−0.18 ± 0.08	0.03
	Minimum temperature on the day of capture	0.25 ± 0.05	0.00
	Relative humidity on the day of capture	0.04 ± 0.01	0.00

## Data Availability

The data presented in this study are openly available in FigShare at https://doi.org/10.6084/m9.figshare.24511504.v1 accessed on 7 November 2023.

## Supplementary Materials

The following supporting information can be downloaded at: https://www.mdpi.com/article/10.3390/insects15010045/s1, Figure S1: Map of the city of Lisbon with the relative location of Lisbon Zoo, Monsanto urban park, the Tagus River, and the meteorological station; Figure S2: Location of adult mosquito traps at Lisbon Zoo; Figure S3: Species accumulation curve of mosquitoes sampled at Lisbon Zoo; Table S1: Weather variables and respective time aggregation used for the GLM models for *Culex pipiens* abundance.
