# Supplementary material for: Mosquito (Diptera: Culicidae) Fauna of a Zoological Park in an Urban Setting: Analysis of Culex pipiens s.l. and Their Biotypes
Source: Insects. 2024 Jan 9;15(1):45. doi: 10.3390/insects15010045 (PMC10816151; doi:10.3390/insects15010045)
Supplement: Supplementary file 1 [file insects-15-00045-s001.zip › insects-2789552-supplementary.pdf]

Supplementary data

Figure S1 – Map of the city of Lisbon with the relative location of Lisbon Zoo (1), Monsanto urban park (2), the Tagus River (3) and the meteorological station (4).

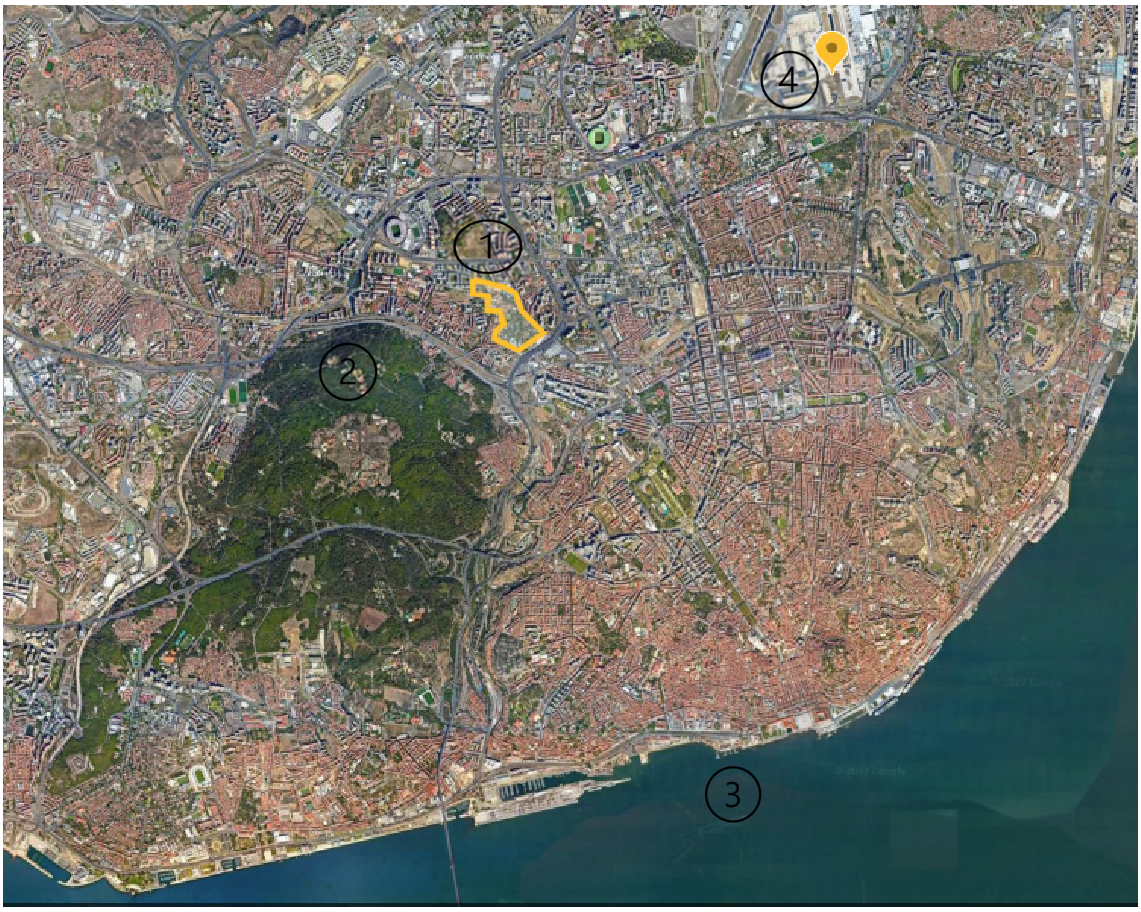

Figure S2 – Location of adult mosquito traps at Lisbon Zoo.

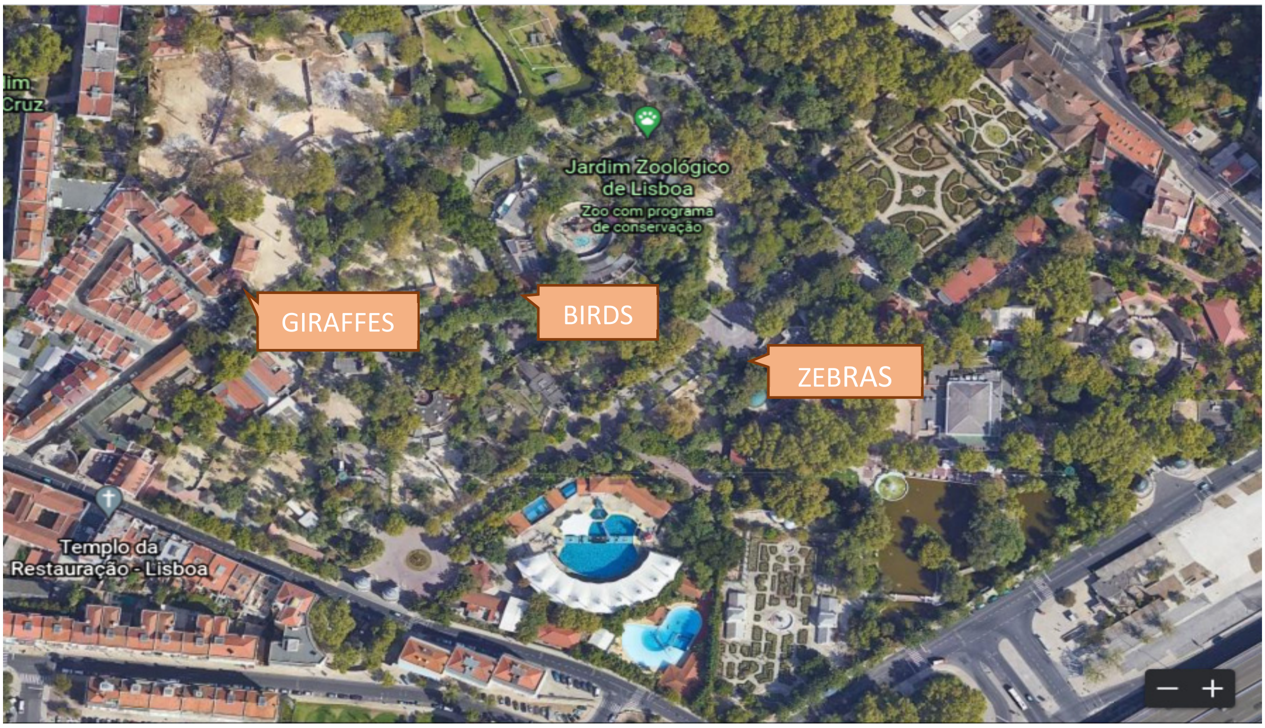

Figure S3 – Species accumulation curve of mosquitoes sampled at Lisbon Zoo.

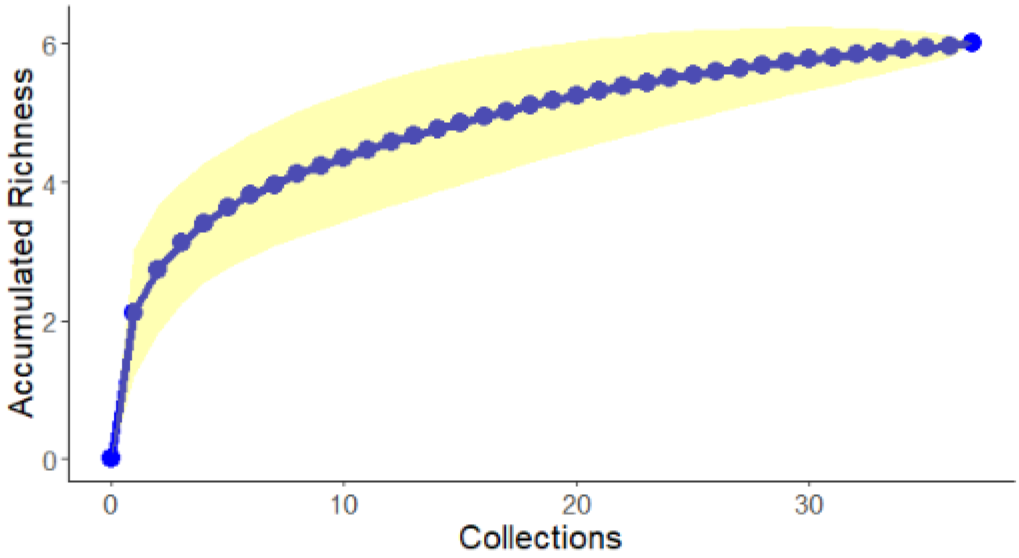

Table S1 - Weather variables and respective time aggregation used for the GLM models for *Culex pipiens* abundance.

Weather variable	*Aggregation*	*Time Lag*
Maximum Temperature	average	Week before capture
	average	Week of capture
	--	Day of capture
Minimum Temperature	average	Week before capture
	average	Week of capture
	--	Day of capture
Average Temperature	average	Week before capture
	average	Week of capture
	--	Day of capture
Precipitation	Yes or no	Day of capture
	average	Week before capture
	average	Week of capture
	Accumulated (sum)	Week before capture
Relative humidity (RH)	average	Week before capture
	average	Week of capture
Wind Intensity	average	Week of capture
	-	Day of capture
